# Is Anything Gained From Estimating Blood Volume in Hemodialysis From Extracellular Volume Divided by a Constant Factor?

**DOI:** 10.1111/hdi.70008

**Published:** 2025-07-07

**Authors:** Sebastian Mussnig, Simon Krenn, David Keane, Manfred Hecking, Daniel Schneditz

**Affiliations:** ^1^ Department of Epidemiology, Center for Public Health Medical University of Vienna Vienna Austria; ^2^ Lilibeth Caberto Kidney Clinical Research Unit London Health Sciences Centre Research Institute London Canada; ^3^ IUVABIT e.U., Analytics & Data Science Vienna Austria; ^4^ CURAM, Center for Research in Medical Devices University of Galway Galway Ireland; ^5^ Division for Nephrology and Dialysis, Department of Medicine III Medical University of Vienna Vienna Austria; ^6^ Otto Loewi Research Center for Vascular Biology, Immunology and Inflammation, Division of Physiology & Pathophysiology Medical University of Graz Graz Austria

To the editor,

In a follow‐up to a previous study [1], Kron et al. recently [2] examined whether blood volume (BV) in hemodialysis patients could be estimated from extracellular volume (ECV) derived from bioimpedance spectroscopy, assuming a constant physiological 1‐over‐3 relationship between these volumes, described previously [1, 3]. The authors compared BV estimated from bioimpedance (ECV/3) to their experimental BV, measured using their dialysate dilution method [4]. The overall bias was 0.2 L and small, but the 95% confidence limits were ±1.2 L and large. The authors attributed all error to bioimpedance and to deviations from the population‐derived 1‐over‐3 relationship, and it ultimately remained unclear how estimated BV information should be used.

Kron et al. asked “Can Bioimpedance Analysis Be Used to Estimate Absolute Blood Volume in Hemodialysis Patients?” and answered that this estimation is only possible in patients without severe fluid overload. This interpretation hinges on data that the authors were not able to provide because they excluded patients with “severe volume expansion” and selected “stable patients without recorded intradialytic morbid events in the previous 3 weeks”. We reproduced the analysis by Kron et al. [2] in a larger population [5] without exclusions, with longitudinal measurements over multiple months. We found (a) that the margin of error might even be larger and showed (b) that BV is overestimated by ECV/3 in those patients who are increasingly fluid overloaded (Figure 1). In a separate cohort with repeated measurements covering 5 weeks, we found that ECV and fluid overload followed an expected weekly cycle, decreasing during the week and increasing during the long treatment interval, whereas pre‐dialysis BV remained more or less unchanged and independent of fluid overload, without any clear weekly pattern [6]. Similar to previous findings [7], our data suggest that variable fluid overload is predominantly located in the extravascular space and that the BV/ECV ratio may not be constant.

**FIGURE 1:**
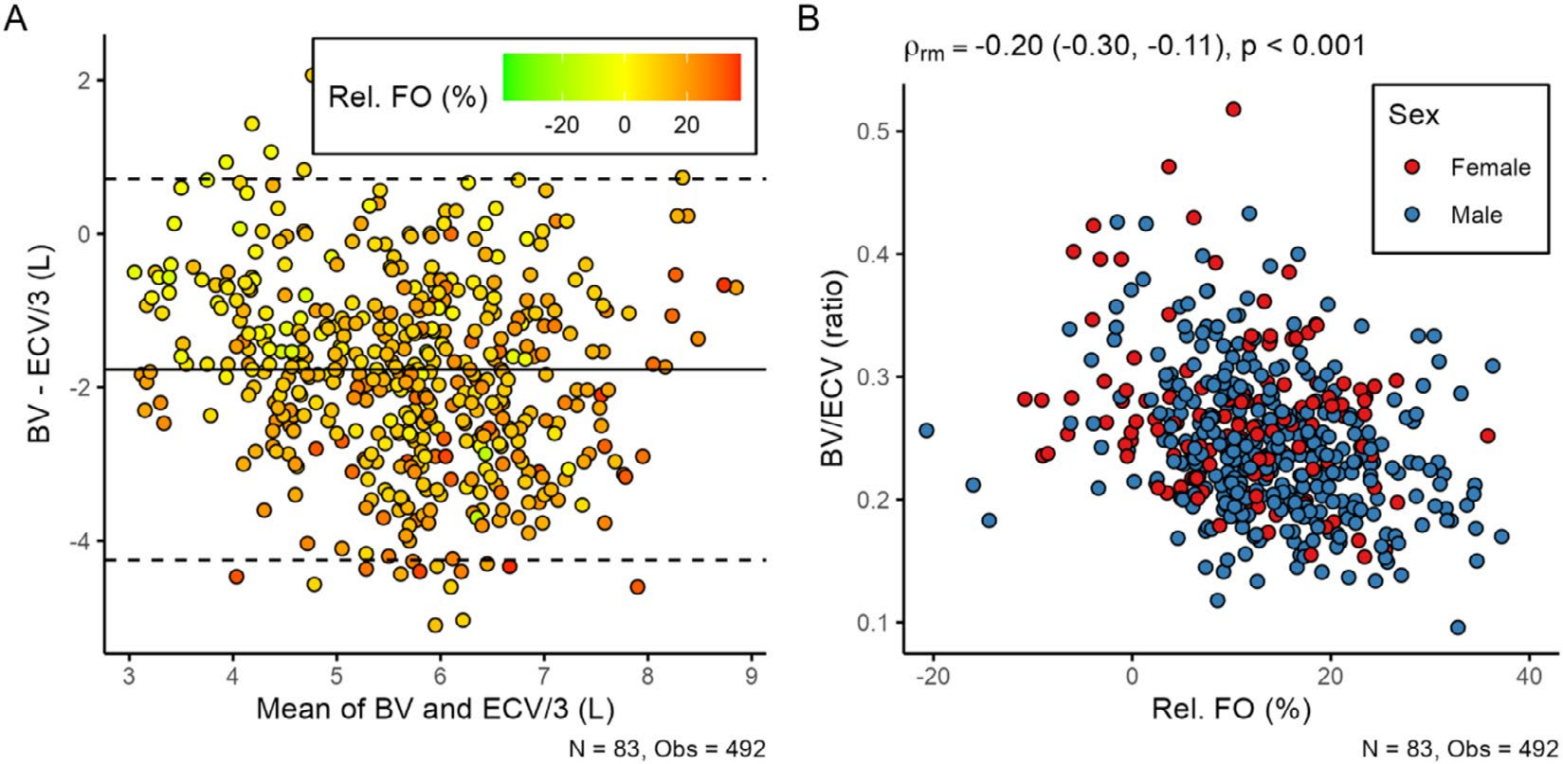
Relationship between dialysate‐dilution‐ and extracellular‐volume‐derived blood volume. We show data from 83 patients on maintenance hemodialysis (N) who received altogether 492 treatments (Obs), spanning 3 months. Blood volume (BV) was measured using dialysate‐bolus dilution, and ECV was measured using pre‐dialysis bioimpedance spectroscopy, as was previously done by Kron et al. [2]. Panel A: Bland–Altman plot of measured BV vs. BV estimated from ECV/3. The bias between BV and ECV/3 was in the range of −2 L with a 95% confidence interval of about 2.5 L. The color code differentiates between various degrees of fluid overload relative to ECV (Rel. FO), as measured by bioimpedance. The bias between BV and ECV/3 increased (became more negative) with increasing Rel. FO. Panel B: Scatterplot showing Rel. FO and the measured BV/ECV ratio. $\rho_{\mathrm{rm}}$ is the repeated‐measures correlation coefficient accounting for repeated measurements per patient. The negative correlation suggests that the BV/ECV ratio decreased with increasing Rel. FO within patients, implying that excess fluid volume was predominantly located in the extravascular space.

While Kron et al. are to be congratulated for their contributions to volume estimation in dialysis patients, the present analysis [2] does not seem appropriate to answer the question they posed not only because data were obtained at a single time point, but more importantly because they excluded fluid overload as the condition of interest. Direct measurement of blood volume is required to uncover pathological imbalances between the intravascular and interstitial spaces. BV should not be estimated by ECV/3 assuming a constant factor in patients who suffer from fluid disequilibrium.

## Conflicts of Interest

The authors declare no conflicts of interest.

## Data Availability

The data that support the findings of this study are available from the corresponding author upon reasonable request.
